# Non-Coding RNAs in Stroke and Neuroprotection

**DOI:** 10.3389/fneur.2015.00050

**Published:** 2015-03-13

**Authors:** Julie A. Saugstad

**Affiliations:** ^1^Department of Anesthesiology and Perioperative Medicine, Oregon Health & Science University, Portland, OR, USA

**Keywords:** cerebral ischemia, neuroprotection, preconditioning, non-coding RNA, microRNA

## Abstract

This review will focus on the current state of knowledge regarding non-coding RNAs (ncRNA) in stroke and neuroprotection. There will be a brief introduction to microRNAs (miRNA), long ncRNAs (lncRNA), and piwi-interacting RNAs (piRNA), followed by evidence for the regulation of ncRNAs in ischemia. This review will also discuss the effect of neuroprotection induced by a sublethal duration of ischemia or other stimuli given before a stroke (preconditioning) on miRNA expression and the role of miRNAs in preconditioning-induced neuroprotection. Experimental manipulation of miRNAs and/or their targets to induce pre- or post-stroke protection will also be presented, as well as discussion on miRNA responses to current post-stroke therapies. This review will conclude with a brief discussion of future directions for ncRNAs studies in stroke, such as new approaches to model complex ncRNA datasets, challenges in ncRNA studies, and the impact of extracellular RNAs on human diseases such as stroke.

## Introduction

Despite advances in preclinical studies that have established mechanisms of cell death in brain ischemia and identified potential strategies to prevent or treat brain injury following stroke, few of these advances have successfully translated into clinical practice. Endogenous neuroprotective responses have been studied in experimental stroke largely using a model wherein a sublethal duration of ischemia is given before a stroke (preconditioning). However, additional methods of preconditioning are also effective in reducing stroke injury, i.e., hypoxia, anesthetic agents, and activation of Toll-like receptors, and natural states of neuroprotection in animals such as hibernation that lend important insight to the topic. More recent efforts have focused on strategies for neuroprotection after a stroke (post-conditioning), which is more relevant to the clinical presentation in human patients. This review will present the current state of knowledge regarding evidence for specific families of non-coding RNAs (ncRNA) in experimental stroke and neuroprotection: microRNAs (miRNA), long non-coding RNAs (lncRNA), and piwi-interacting RNAs (piRNA). The literature search was performed using PUBMED queried with a combination of terms listed in Table 1, and the number of references returned for each query.

**Table 1:** **Literature search strategy**.

Search terms			Number of references
Ischemic preconditioning	Cerebral	Review	104
Neuroprotection	Cerebral ischemia	MiRNA	10
Preconditioning	Cerebral ischemia	MiRNA	5
	Stroke	MiRNA	4
Post-conditioning	Cerebral ischemia	MiRNA	0
	Stroke	MiRNA	1
MiRNA	Stroke		78
	Ischemia		147
	Preconditioning		23
	Neuroprotection		28
	Neuroprotection	Ischemia	12
	Neuroprotection	Cerebral ischemia	46
	Neuroprotection	Cerebral ischemia	10
		Ischemic stroke	9
	Biomarker	Cerebral ischemia	2
	Biomarker	Stroke	9
PiRNA	Brain		9
	Stroke		1
	Ischemia		1
	Preconditioning		0
	Neuroprotection		0
	Neuroprotection	Ischemia	0
		Cerebral ischemia	1
	Neuroprotection	Cerebral ischemia	0
	Neuroprotection	Ischemic stroke	0
LncRNA	Brain		56
	Stroke		2
	Ischemia		3
	Preconditioning		0
	Neuroprotection		0
	Neuroprotection	Ischemia	0
	Cerebral ischemia		2
Exosome	MiRNA	Cerebral ischemia	0
		Cerebral ischemia	0
	Stroke		4
Post-stroke	MiRNA		1

*PUBMED was queried with the following combination of search terms, and the resultant numbers of references returned for each combination are listed*.

In recent years, there has been increasing focus on the role of ncRNAs as regulators of post-transcriptional gene expression, and of responses to ischemia and neuroprotection (1). Spinal cord injury and traumatic brain injury also lead to changes in miRNA expression that precedes injury (2). Further, dysregulation of ncRNA activities has been closely linked to the pathophysiology of cerebral vascular endothelium and neurologic functional disorders in the brain’s response to ischemia (3). Experimental stroke models have been used for global profiling of brain or blood miRNAs in animals, or to manipulate specific miRNAs or their targets to examine their potential as neuroprotective strategies for stroke. Given the current published reviews for the role of miRNAs in the occurrence and development of post-stroke depression (4), and in the regulation of miRNAs that lead to angiogenesis and remyelination (5), neurogenesis (6), and spontaneous recovery (7), they will not be discussed here. Also, outside the scope of this review are many miRNAs studies on cardiac ischemia and protection that may provide important insights on miRNAs in cerebral ischemia and neuroprotection (8).

### Non-coding RNAs

Early biochemistry studies identified three families of RNA that function cooperatively in the process of protein synthesis: messenger, transfer, and ribosomal RNA. Messenger RNAs (mRNA) carry genetic information copied from DNA in a series of three-base codes that specify a particular amino acid, specifying the polypeptide sequence. Transfer RNAs (tRNA) carry and bind a complementary amino acid to the growing end of a polypeptide chain. Ribosomal RNAs (rRNA) bind to protein complexes, which physically move along mRNAs and catalyze the assembly of amino acids into polypeptide chains (9). Additional families of ncRNAs that play essential roles in the processes of protein synthesis were subsequently discovered (10). For example, small nuclear RNAs (snRNA) bind to proteins to form spliceosomes that process pre-mRNA into mature mRNA in the nucleus (11), while small nucleolar RNAs (snoRNA) guide chemical modifications of other RNAs (12). This review will focus on three newer families of ncRNAs; (1) miRNAs that regulate post-transcriptional gene expression, largely by direct effects on mRNAs (13), (2) lncRNAs that can regulate transcription and translation, and act as epigenetic modifiers, and (3) piRNAs that largely exert effects on transcription and genomic maintenance. The reader is directed to a 2014 Frontiers in Cellular Neuroscience Research Topic entitled “*Regulatory RNAs in the Nervous System*” for comprehensive discussions regarding the biosynthesis and function of these ncRNAs, as well as a recent review entitled “*Non-Coding RNAs as Potential Neuroprotectants against Ischemic Brain Injury*,” which beautifully depicts the current understanding of the biogenesis pathways for each of these three ncRNAs discussed herein (14).

#### MicroRNAs

The miRNAs are the most well-characterized family of ncRNA to date. Mature, functional miRNA sequences are ~21–23 nucleotides (nt) in length, and are most recognized for their roles in the regulation of post-transcriptional gene expression via direct effects on the 3′-untranslated region (3′UTR) of mRNAs that lead to translational repression or mRNA degradation. MiRNA biogenesis has been previously described and will not be presented here, but the reader is guided to a recent comprehensive review of this topic (15). The miRNAs are particularly abundant in the nervous system where they serve as effectors of brain development (16–18) and maintenance of the neuronal phenotype (19, 20). MiRNAs also drive the maturation of dendrites and spines (21–23) and serve as effectors of synaptic plasticity and function (24–30). Emerging studies highlight roles for miRNAs in extracellular vesicles (EVs) where they contribute to cell–cell communication in the brain (31–33) and throughout the nervous system (34). As such, dysregulation of EVs may underlie diverse neurological disorders including brain tumors (35), neurodegeneration, and neuroinflammation (34), and EVs in biofluids are currently being evaluated for their potential as biomarkers or therapeutic strategies for the treatment or prevention of human diseases, including many brain disorders (36).

#### Long non-coding RNAs

The lncRNAs are >200 nt in size, and they differ greatly from each other with respect to their size, interacting partners, and modes of action (37). The lncRNAs are processed through pathways similar to those of protein-coding genes, and have similar histone-modification profiles, splicing signals, and exon/intron lengths. The complex intergenic, overlapping, and antisense patterns of lncRNAs relative to adjacent protein-coding genes suggests that many lncRNAs regulate the expression of adjacent protein-coding genes. LncRNAs are also implicated in the regulation of transcription and post-transcriptional gene expression, as comprehensively reviewed (38). LncRNAs can stabilize or promote the translation of target mRNAs through extended base pairing, or they can inhibit target mRNA translation or facilitate mRNA decay via partial base pairing. Even in the absence of any base pair complementarity, lncRNAs can suppress precursor mRNA splicing and translation by acting as decoys for RNA-binding proteins or miRNAs, or compete for miRNA-mediated inhibition of mRNAs resulting in increased expression of corresponding proteins. Over one-half of all lncRNAs are expressed in the brain (39) and they regulate many CNS processes (40). Hence, dysregulation of lncRNAs can also contribute to CNS pathologies such as neurodegeneration, neuroimmunological disorders, primary brain tumors, and psychiatric diseases (41). As such, lncRNAs present new targets for the understanding, diagnosis, and treatment of CNS disorders (42).

#### Piwi-interacting RNAs

The piRNAs are ~25–33 nt in length, depending on the PIWI protein group they bind to, and they lack sequence conservation between organisms. Recent reviews highlight the complexity of piRNA biogenesis pathways, which have just begun to be elucidated (43, 44). PiRNA biogenesis pathways are complicated as they are distinct in individual organisms, and they are distinct from miRNA pathways in that there is no evidence for a double-stranded RNA precursor and biogenesis is independent of Dicer (45–47). There are two proposed pathways for generating piRNAs: a primary processing pathway and a “ping-pong” amplification loop, as recently reviewed (14). PiRNA clusters are transcribed in the sense or antisense direction, and the long single-stranded RNA serves as the basis for piRNA production. The piRNAs protect germline cells from transposons in organisms as they guide PIWI proteins to complementary RNAs derived from transposable elements, and then the PIWI proteins cleave transposon RNA. Transposons are genetic elements that copy themselves to RNA, then back to DNA, and then integrate back into the genome where they may induce mutations that cause disease by inserting near or within other genes to disrupt their function. The piRNAs have been dubbed as the guardians of the genome because of their ability to silence deleterious retrotransposons (48). Very recent studies also show piRNAs as effectors in the epigenetic control of long-term memory storage in the brain (49).

Together, these three newer families of ncRNAs serve to regulate many aspects of cellular biology that ultimately control phenotype, function, and genomic integrity. As such, dysregulation of any or all of these ncRNAs will likely have profound impact on CNS disorders.

## Ischemia

### Overall effect of ischemia on miRNA expression

The overall effect of stroke on miRNA expression in brain and blood has been examined in experimental rodent models and in human subjects. Most rodent studies have used transient middle cerebral artery occlusion (MCAO) to induce focal cerebral ischemia. The first such study used microarray analysis to examine overall expression of miRNAs in rat brain and blood at 24 and 48 h after ischemia and showed that miRNAs are regulated by focal ischemia and may potentially serve as blood biomarkers for ischemia (50). Focal ischemia studies in spontaneously hypertensive rats also showed altered miRNA profiles at all reperfusion times examined (3 h to 3 days), with several miRNAs altered at multiple time points (51). Bioinformatic analysis predicted mRNA targets of the ischemia-regulated miRNAs as proteins known to mediate inflammation, transcription, neuroprotection, receptor function, and ionic homeostasis (51). Further, *in silico* analysis of 8 ischemia-induced miRNAs revealed sequence complementarity to 877 gene promoters, suggesting that ischemia-regulated miRNAs directly modulate gene expression. Importantly, these studies also showed that focal ischemia did not alter mRNA levels for Drosha, Dicer, Pasha, and Exportin-5, proteins involved in the miRNA biogenesis pathway (51). Studies focused on humans revealed that circulating blood miRNA profiles were altered in young ischemic stroke patients (18–49 years) and that specific miRNAs that could classify subtypes of stroke (large-vessel atherosclerosis vs. small-vessel disease vs. cardioembolism) were still detectable in blood several months after injury (52). The first RNAseq studies identified significant changes in 78 known and 24 novel miRNAs in rat brain following ischemia, and Kyoto Encyclopedia of Genes and Genomes (KEGG) pathway analysis predicted that protein targets of these miRNAs function in signaling transduction, MAPK signaling, NF-kappaB signaling, and neurotrophin signaling (53).

Together, these studies show that miRNAs are regulated in brain and blood where they likely regulate translation, and transcription, in post-ischemic brain. These studies also established the potential for blood miRNAs to be developed as clinical biomarkers for the diagnosis and prognosis of ischemic stroke in humans.

### Sex-specific miRNA responses to ischemic injury

Females are more resistant to stroke injury than males until menopause (54), and responses to experimental stroke and cell death are sexually dimorphic (55). In males, stroke initiates mitochondrial release of apoptosis-inducing factors that result in caspase-independent cell death, while in females stroke primarily triggers mitochondrial cytochrome c release and subsequent caspase activation. At baseline, females have higher levels than males of brain mRNA for X-linked inhibitor of apoptosis (XIAP), the primary endogenous inhibitor of caspases, and stroke significantly decreases XIAP mRNA in females but not in males. However, XIAP protein levels are decreased in both sexes after stroke, likely due to different levels of miR-23a, which binds to the XIAP 3′UTR, in male and female ischemic brain (55). Subsequent studies also revealed distinct differences in miRNA responses to ischemia in male and female brain, as well as a signature miRNA response to ischemia common to males and females (56). The finding that there are sex differences in miRNA expression during development in rat cortex may bear on differential responses to ischemia in adults (57). Accordingly, a recent study investigated the impact of age and sex on miRNA expression in adult (6 months) and middle-aged (11–12 months) female and male rats subjected to the endothelin-1 model of MCAO (58). Consistent with MCAO injury, infarct volume and sensory-motor deficits were significantly reduced in adult females compared with middle-aged females, adult males, or middle-aged males. Analysis of blood at 2 days post-stroke revealed 21 differentially regulated circulating miRNAs, and principal component analysis demonstrated that most of the variance was due to age. At 5 days post-stroke, analysis of blood revealed 78 differentially regulated circulating miRNAs, and principal component analysis confirmed that most of the variance was associated with sex. Brain miRNAs analyzed at 5 days post-stroke revealed a small cohort of miRNAs (miR-15a, miR-19b, miR-32, miR-136, and miR-199a-3p) highly expressed exclusively in adult females. These patterns of circulating miRNA expression suggest that age is the primary influence on the initial severity of stroke pathology, but sex is the primary influence on recovery from stroke. Together, these studies support that miRNA expression can be different between males and females, which may contribute to sexually dimorphic responses to ischemia.

### Effect of ischemia on specific miRNAs and targets

Subsequent studies began to investigate the functional significance of individual ischemia-regulated miRNAs on the molecular mechanisms that lead to brain cell death. In addition to those described above, several of these specific miRNAs are discussed below.

#### miR-497

The expression of miR-497 was significantly induced in mouse brain 24 h after MCAO, and in mouse N2A neuroblastoma (N2A) cells after *in vitro* ischemia induced by oxygen-glucose deprivation (OGD) (59). Yin and colleagues showed a direct correlation between miR-497 and cell death: gain or loss of miR-497 promoted or decreased OGD-induced neuronal cell loss, respectively. Luciferase assays showed direct binding of miR-497 to the 3′UTR of B-cell lymphoma 2 (Bcl-2) and Bcl-w mRNAs, whose proteins function as key regulators in attenuating stroke-induced apoptotic cell death (60–63). Inhibition of miR-497 in mouse brain enhanced Bcl-2/-w protein expression in the ischemic region, attenuated ischemic brain infarction, and improved neurological outcomes in response to focal ischemia.

#### miR-29b

Subsequent studies showed that miR-29b promoted ischemic cell death by targeting Bcl-2. MiR-29b expression was significantly increased in rat brain by focal ischemia, and in primary cultured neurons by OGD (64). Cell death was also directly correlated with miR-29b expression: gain or loss of miR-29b promoted or decreased OGD-induced neuronal cell loss, respectively. Additionally, miR-29b directly bound to the 3′UTR of Bcl-2 mRNA, and overexpression of the *bcl-2* gene rescued neuronal cell death induced by miR-29b.

#### miR-134

Overexpression of miR-134 promoted hippocampal cell death in cultured neurons subjected to OGD, while inhibition of miR-134 was protective. Expression of cyclic AMP (cAMP) response element-binding protein (CREB), a putative target of miR-134, was inversely regulated by miR-134 (65).

#### miR-21

*In situ* hybridization showed increased expression of miR-21 in rat neurons in the ischemic boundary zone at 2 and 7 days after embolic MCAO, and RT-qPCR studies verified a quantitative increase in mature miR-21 in neurons isolated from the ischemic boundary zone (66). Overexpression of miR-21 protected cortical neurons from cell death induced by OGD, while inhibition of miR-21 promoted OGD-induced cell death. Overexpression of miR-21 in neurons significantly reduced levels of Fas Ligand (FASLG), a cell death-inducing ligand, and luciferase assays showed that miR-21 binds to the 3′UTR of FASLG mRNA.

#### miR-347

Permanent focal cerebral ischemia in rat brain also leads to regulation of miRNAs between 3 h and 14 days following MCAO (67). Of particular interest was miR-347 that was upregulated at both acute and late phases following ischemia. Overexpression of miR-347 increased neuronal apoptosis and induced the expression of novel mRNA targets including acyl-CoA synthetase long-chain family member 4 (Acsl4), Bcl-2/adenovirus E1B 19 kDa protein-interacting protein 3-like (Bnip3l), and phytanoyl-CoA 2-hydroxylase interacting protein (Phyhip) (67).

These independent studies demonstrate that although there are diverse miRNAs regulated by ischemia, multiple miRNAs act directly and indirectly on cell-death proteins and can influence both pro- and anti-apoptotic pathways.

### Temporal and regional expression of specific miRNAs in ischemia

MiRNAs can induce distinct outcomes in a temporal- and cell-specific manner. Cultured rat cortical neurons and astrocytes subjected to OGD exhibit unique miRNA expression patterns. OGD increased miR-29b expression in neurons by twofold after 6 h and by fourfold after 24 h compared to miR-21 that remained unchanged until after 24 h of OGD (68). However, OGD increased miR-29b and miR-21 in astrocytes only after 12 h, and further, altered expression of miR-30b, miR-107, and miR-137 was specific to astrocytes. Responses to ischemia in mouse brain also showed increased expression of miR-181 in the core, where cells die, and decreased expression of miR-181 in the penumbra, where cells survive (69). Reduction in miR-181a was correlated with increased levels of glucose-regulated protein, 78 kDa (GRP78), a heat shock protein that functions within the chaperone network to increase resistance to and improve recovery from ischemic brain injury (69, 70). Further studies compared miRNA expression induced by OGD in rat cortical neurons to *in vivo* miRNA expression in mice exposed to the three-vessel occlusion model of ischemia (71). These studies showed that while multiple miRNAs were differentially regulated by ischemia in each model, miR-19b, miR-29b-2*, and miR-339-5p were all significantly upregulated in common to both models (71). These studies suggest that there are signature miRNA responses to ischemia in salvageable brain regions, information that may lend new insights into cell- and temporal-specific protective strategies for the treatment of ischemia.

### lncRNAs in ischemia

The effect of ischemia on lncRNA expression was first examined in adult male spontaneously hypertensive rats at 3, 6, and 12 h after MCAO (72). The results showed that of 8314 lncRNAs analyzed, 443 were significantly changed at all time points (359 upregulated and 84 downregulated). Of these, 61 stroke-responsive lncRNAs showed >90% sequence homology with exons of protein-coding genes, but *in vitro* translation of these lncRNAs did not form any protein products (72). Based on the roles of lncRNAs in cellular homeostasis during development and disease via interactions with chromatin-modifying proteins (CMPs), the effect of ischemia on the association of lncRNAs with CMPs, Sin3A, and corepressors of the RE1 (Repressor Element 1)-silencing transcription factor (coREST) was examined (73). These studies revealed that 177 of the 2497 lncRNAs in rat cerebral cortex showed increased binding to either Sin3A or coREST following ischemia, and that of these, 26 lncRNAs enriched with Sin3A and 11 lncRNAs enriched with coREST were upregulated by ischemia. Thus, these studies suggest that ischemia-induced lncRNAs might associate with CMPs to modulate the post-ischemic epigenetic landscape.

### piRNAs in ischemia

The first evidence for expression of piRNAs in the nervous system was revealed by RNAseq analysis of mouse hippocampal RNA (74). PiRNA expression in adult male spontaneously hypertensive rats examined 24 h after focal ischemia revealed that of ~40,000 piRNAs analyzed, 105 piRNAs were significantly altered in cerebral cortex with 54 upregulated and 51 downregulated (75). Bioinformatic analysis of four of the top stroke-responsive piRNAs (two up, two down) revealed that their transposon targets belong exclusively to the class of retrotransposons. Further, analysis of the promoter region of 10 stroke-responsive piRNAs revealed binding sites for 159 transcription factors, including the zinc finger, Kruppel, and E2F transcription factor-like families, supporting that transcription factors control the expression of stroke-responsive piRNAs.

## Neuroprotection

### Preconditioning

The studies discussed here will focus only on miRNA responses to preconditioning models that lead to neuroprotection against stroke as there are no published studies regarding the impact of post-conditioning on miRNA, lncRNA, or piRNA in cerebral ischemia to date. Preconditioning is a phenomenon whereby a brief, non-lethal duration of a stimulus induces a transient state of resistance (tolerance) to a subsequent lethal duration of a stimulus. Ischemic preconditioning (IPC) is an experimental technique induced by a short duration of ischemia that protects the brain from a subsequent injurious duration of ischemia (76, 77). New protein synthesis is required for IPC-induced tolerance (78), and the signature of ischemic tolerance is global repression of gene expression (79–81). However, additional preconditioning stimuli such as hypoxia, anesthesia, and activation of the Toll-like receptors can induce tolerance and protection against ischemia, as discussed below.

#### Ischemic preconditioning

Ischemic tolerance was first examined in a gerbil model of global cerebral ischemia (82, 83). The first studies to examine miRNA responses to ischemia in mice evaluated their expression in response to IPC (15 min MCAO), ischemia (60 min MCAO), and tolerance (15 min MCAO, 72 h reperfusion, 60 min MCAO). Microarray analysis of cortical tissue isolated from adult male mice 24 h after each treatment revealed that miRNAs were regulated by each treatment with 192 upregulated and 95 downregulated by IPC, and that there was very little overlap in the miRNAs regulated between all three treatment groups (84). Bioinformatic analysis focused on those miRNAs downregulated by IPC that could result in increased protein synthesis revealed that predicted targets included transcriptional regulators such as methyl cytosine-phosphate-guanine (CpG) binding protein 2 (MeCP2) (84). MecCP2 is a global transcriptional regulator that largely serves to repress gene expression but can also activate gene expression (85). MeCP2 protein, but not mRNA expression, increased in mouse cortex by 8 h after IPC, and MeCP2 knockout mice showed increased sensitivity to the preconditioning stimulus and were not tolerant to a subsequent ischemic event (84). Recent studies in a cardiac ischemia model showed that mesenchymal stem cells, which protect ischemic cardiomyocytes by secretion of paracrine factors, secreted exosomes enriched with miR-22 following IPC, and uptake of these exosomes by cardiomyocytes reduced ischemia-induced apoptosis via direct targeting of MeCP2 (86). These studies support a protective role for MeCP2 in both cerebral and cardiac ischemia, although the specific gene targets in each model need to be identified.

Analogous studies to examine miRNA expression in a rat model of IPC induced in adult male spontaneously hypertensive rats by 10 min MCAO revealed significant changes in 51 miRNAs (26 upregulated and 25 downregulated) at 6 h of reperfusion, and 20 of these miRNAs remained altered 3 days after IPC (87). Bioinformatic analysis of the miRNAs and KEGG Pathway analysis of their predicted targets showed MAP-kinase and mammalian target of rapamycin (mTOR) signaling as the top two pathways targeted by the upregulated miRNAs, and Wnt and gonadotropin releasing hormone (GnRH) signaling as the top two pathways targeted by the downregulated miRNAs. Interestingly, these studies also showed that multiple miRNAs downregulated by IPC were predicted to target MeCP2 in rat, consistent with previous studies on IPC in mouse (84).

Additional studies in adult male mice subjected to IPC or ischemia revealed 8 miRNAs that together comprise the miR-200 and miR-182 families of miRNAs that were selectively upregulated 3 h after IPC, and 2 miRNAs (miR-681 and miR-197) that were selectively upregulated 24 h after IPC (88). No miRNAs were downregulated at 3 h after IPC, and only 1 miRNA (miR-468) was downregulated at 24 h after IPC. Transfection of the 8 IPC upregulated miRNAs into N2A cells revealed 5 miRNAs that increased cell survival in response to OGD, and decreased expression of prolyl hydroxylase 2 (PHD2), a predicted target of the miR-200 family involved in the ubiquitin/proteosomal pathway. Reduced levels of PHD2 protein were also detected 24 h after IPC, but not after ischemia.

#### Hypoxic preconditioning

Hypoxic preconditioning (HPC) induced by autohypoxia (acute and repetitive exposure to progressive hypoxia) in adult male mice significantly decreased ischemic injury by mechanisms involving multiple conventional protein kinase C βII (cPKCβII)-interacting proteins (89). Bioinformatic analysis of 17 miRNAs (4 upregulated and 13 downregulated) in mouse cortex between HPC and ischemia predicted that cPKCβII, γ, and novel protein kinase Cε (nPKCε)-interacting proteins as targets of the HPC-specific miRNAs (90). Together, these studies support that HPC-induced protection against ischemia involves regulation of cPKC proteins and modulation of calcium signaling in mouse brain.

#### Anesthetic preconditioning

Several experimental studies show that inhalational anesthetics serve as preconditioning agents that lead to neuroprotection against stroke. Administration of isoflurane, halothane, sevoflurane, desflurane, nitrous oxide, and other agents is protective in the setting of stroke, and potential mechanisms of anesthetic preconditioning include antagonism of AMPA and NMDA receptors and reduced excitotoxicity, modulation of cerebral catecholamine release, direct anti-oxidant effects of anesthesia, and reduction of the cerebral metabolic rate (91). Recent studies to examine the contribution of miRNAs to anesthetic preconditioning revealed that isoflurane significantly increased expression of miR-203 in rat B35 neuron-like cells, and there was a trend toward increased miR-203 expression in rat cerebral cortex after isoflurane exposure (92). Overexpression of miR-203 in B35 cells increased both tolerance to ischemia and expression of Phospho-Akt, a protein kinase that promotes cell survival. The effect of sevoflurane preconditioning on rat brain miRNA expression following transient cerebral ischemia revealed 3 upregulated and 9 downregulated miRNAs (93). Among these, miR-15b expression was increased after ischemia, while sevoflurane preconditioning downregulated miR-15b expression and increased expression of Bcl-2, a target of miR-15b. These studies suggest that sevoflurane may exert anti-apoptotic effects by inhibiting miR-15b expression leading to increased expression of Bcl-2.

#### Toll-like receptor preconditioning

Preconditioning can be induced by activation of the Toll-like receptors (94, 95). CpG oligodeoxynucleotide is a Toll-like receptor 9 agonist that when administered 72 h prior to ischemia can reduce injury in rodent and non-human primate models of experimental stroke (96, 97). MiRNA expression studies in adult male mice revealed that miRNAs are not regulated at 3, 24, or 72 h after CpG preconditioning (98), suggesting that alterations in miRNA expression in the brain are not necessary for the neuroprotection induced by systemic CpG preconditioning. Interestingly, CpG-preconditioned mice had fewer total miRNAs regulated in response to MCAO compared with saline-treated mice (57 vs. 73 miRNAs), suggesting that CpG preconditioning alters the miRNA response to stroke (98). Subsequent analysis of the miRNAs differentially expressed in CpG-preconditioned and saline-treated mice at 24 h after stroke revealed that miRNA suppression correlated with the upregulation of genes involved in neuroprotection, suggesting that suppression of differential miRNAs in CpG preconditioning may reprogram the genomic response to stroke to favor a protective outcome.

#### Hibernation torpor

Torpor is a form of sleep that helps animals conserve valuable resources in times of stress, such as in cold or very hot, dry weather. Hibernation torpor (HT) is used to model endogenous mechanisms of tolerance, as metabolic rates decrease in order to conserve energy in both conditions. HT induces massive global SUMOylation in ground squirrels, and overexpression of Ubc9, SUMO-1, or SUMO-2/3 is protective against ischemic injury in cortical neurons subjected to OGD, and in mouse brain (99, 100). SUMO conjugation and global protein conjugation by ubiquitin-like modifiers (ULMs) including NEDD8, ISG15, UFM1, and FUB1 significantly increased in ground squirrel brain during HT. MiRNA analysis of ground squirrel brain during HT revealed that the miR-200 and miR-182 families were among the most consistently depressed miRNAs relative to active animals (101). Inhibition of these miRNA families in SHSY5Y cells resulted in increased global protein conjugation and tolerance to OGD-induced cell death (101). These studies link endogenous tolerance to brain ischemia in hibernating animals to the regulation of a broad range of ULMs via repression of miRNAs.

### Neuroprotection via manipulation of miRNAs and/or their targets

The previous studies support that manipulation of specific miRNAs or their targets altered by IPC or ischemia may be translated into viable neuroprotective strategies for stroke. Identified targets thus far include epigenetic modifiers, heat shock proteins, ubiquitin modifiers, DNA repair and cell cycle proteins, autophagosome assembly proteins, and mediators of inflammatory responses to injury in astrocytes.

#### miR-29

Previous studies showed that miR-29 is highly expressed in adult rat brain, and is significantly downregulated by focal ischemia (51). Bioinformatic analysis revealed DNA (cytosine-5)-methyltransferase 3a (DNMT3a) as a target of miR-29c, suggesting that increased DNMT3a expression in ischemic brain may contribute to stroke injury (102). Indeed, treatment with premiR-29c was protective against ischemia both *in vitro* (PC12 cells) and *in vivo*, suggesting that strategies to replace miR-29c may decrease cell death following ischemia. In addition, the miR-29c gene promoter region contains binding sites for REST, and treatment of PC12 cells with REST siRNA prevented ischemia-induced downregulation of miR-29c, which maintained repression of DNMT3a resulting in significantly reduced ischemic cell death (102).

#### miR-132

Studies show that miR-132 is a neuron-specific miRNA that contains a RE1 site within its promoter regions in mouse and is a validated target of REST in mammalian cells (19). Transient global ischemia markedly decreased miR-132 expression in rat hippocampal CA1 cells at 24 and 48 h after ischemia, due to ischemia-induced enrichment of REST at the miR-132 promoter and repressed expression of miR-132 (103). REST-dependent silencing was specific to miR-132, as REST did not alter expression of miR-9 or miR-124a that also has RE1 sites in their promoter regions. In addition, the repression of miR-132 was selectively induced in vulnerable CA1 neurons, but not in CA3 neurons, supporting that REST-dependent repression of miR-132 is critical to ischemia-induced neuronal death (103). Depletion of REST *in vivo* blocked ischemia-induced loss of miR-132 in hippocampal neurons, while overexpression of miR-132 *in vitro* and *in vivo* afforded robust protection against ischemia-induced neuronal death. These studies support that REST-dependent repression of miR-132 induces neuronal death in global ischemia.

#### miR-181b and miR-134

The expression of miR-181b and miR-134 is decreased by HPC and ischemia in adult mouse cortex, but increased in tolerant brain (90). Targets for miR-181b include heat shock protein A5 (HSPA5) and ubiquitin carboxyl-terminal hydrolase isozyme L1 (UCHL1), and expression of miR-181b in N2A cells repressed HSPA5 and UCHL1 and protected cells against OGD-induced cell death. Further, miR-181b antagomirs reduced caspase-3 cleavage and neuronal cell loss in ischemic cortex and improved neurological deficits in mice after ischemia (104). A subsequent study showed that downregulation of miR-134 in mouse N2A cells is protective against OGD by upregulating heat shock protein A12B (HSPA12B) (105). HSPA12B was validated as a target of miR-134, and increased expression of HSPA12B correlated with attenuation of neural cell damage in OGD-treated N2A cells, and reduced infarct size and improved neurological outcomes in mice with ischemic stroke (4).

#### miR-30a

Downregulation of miR-30a following ischemia in N2A cells and in mouse brain correlated with an increased conversion ratio of LC3 (microtubule-associated protein 1 light chain 3)-II/LC3-I and increased expression of Beclin 1, which are required for formation of the autophagosome (106).

#### miR-124

Ischemia leads to reduced expression of miR-124 in rat brain (107, 108) and downregulation of miR-124 in rats subjected to focal ischemia correlated with increased expression of Ku70, a DNA repair protein. *In vivo* administration of a miR-124 antagomir into rat brain increased expression of Ku70 mRNA and protein in the ischemic region, resulting in reduced cell death and infarct size, and improved neurological outcomes in rat (109).

#### miR-424

Expression of miR-424 is decreased in the plasma of patients with acute ischemic stroke, and in mouse plasma and brain after ischemia (110). Overexpression of miR-424 decreased infarct size and brain edema due to MCAO when administered either pre- or post-treatment. MiR-424 mimics reduced mRNA and protein levels of cell division cycle 25 homolog A (CDC25A), cyclin D1, and cell division protein kinase 6 (CDK6) in BV2 microglial cells, all of which are upregulated in ischemic brain (110).

#### miR-103-1

Recent studies examined miR-103-1 as a treatment strategy for stroke, based on its potential to regulate the expression of the Na^+^/Ca^2+^ exchanger (NCX1), which mediates bidirectional flux of calcium and sodium across the synaptic membrane and increases ischemic damage in rat brain (111). NCX1 was validated as a target of miR-103-1 in cortical neurons, and intracerebroventricular infusion of anti-miR-103-1 not only prevented NCX1 reduction induced by stroke but also significantly reduced the extent of brain ischemia when infused 24 h before stroke onset (112).

#### Astrocyte miRNAs

Ischemic brain extract contains factors that elicit increased expression of stromal cell-derived factor-1 (SDF-1), a chemokine that is induced by proinflammatory stimuli and that activates leukocytes, in C6 cells and primary astrocytes (113). SDF-1 expression increased in preconditioned astrocytes contributes to brain tolerance via the regulation of the miR-223/miR-27b pathways (113). Neuroprotection by manipulation of miRNA-related pathways are also found in astrocyte-rich miRNAs, including miR-181 and miR-29 families, and miR-146a, and their validated targets, GRP78, and Bcl-2 family members (114).

### Effects of post-stroke neuroprotective therapies on miRNAs

Recent studies show that post-stroke therapies can effectively regulate the expression of miRNAs and their targets, and these miRNAs may serve as direct targets for clinical intervention in stroke. For example, post-stroke treatment of rats with valproic acid, a histone deacetylase inhibitor and a mood stabilizer, upregulated the expression of several miRNAs including miR-331 and miR-885-3p, and improved neurological deficits and motor performance following ischemia (115). In addition, studies show that using Velcade (a proteasome inhibitor that may also suppress TLR signaling) administered in combination with tissue plasminogen activator (tPA) is neuroprotective against ischemia in aged rats (116). The combination of Velcade plus tPA resulted in increased levels of miR-l46a on cerebral endothelial cells, which express TLRs, and outcomes were associated with reduced expression of vascular interleukin-1 receptor-activated kinases 1 (116). Further, it is well known that ischemic injury is more severe in older animals as compared to younger animals, and this difference is associated with reduced availability of insulin-like growth factor-1 (IGF-1) whose levels decrease with age. IGF-1 infusion following stroke, which prevents estrogen neurotoxicity in middle-aged female rats, altered the expression of two conserved IGF pathway regulatory miRNAs, Let-7f, and miR-1. Adult female rats treated with anti-miR-1 as late as 4 h after ischemia had significantly reduced cortical infarct volume, and anti-Let-7 robustly reduced both cortical and striatal infarcts and preserved sensorimotor function and inter-hemispheric neural integration (117). However, anti-Let-7 was only effective in intact females and had no effect on males or ovariectomized females, indicating that the gonadal steroid environment critically modifies miRNA action (117). Further studies examined the effect of post-stroke IGF-1 treatment on miRNA expression in middle-aged female rats (118). IGF-1 administered immediately after ischemia significantly reduced infarct volume in 9- to 11-month female rats 24 h after stroke. Post-stroke IGF-1 treatment significantly downregulated 8 out of 168 disease-related miRNAs in tissue isolated 4 h after ischemia. KEGG pathway analysis predicted that targets of these miRNAs include proteins involved in PI3K–Akt signaling, cell adhesion/ECM receptor pathways, and T-and B-cell signaling. Further, Phospho-Akt was reduced by 4 h after ischemia, but was elevated by IGF-1 treatment at 24 h after ischemia. IGF-1 also induced Akt activation, an effect that was preceded by a reduction of BBB permeability and global suppression of cytokines at 4 h post-stroke (118).

## Conclusion

The goal of this review was to present the current state of knowledge regarding ncRNAs and their role(s) in stroke and neuroprotection. However, of these three relatively new families of ncRNAs, miRNAs are the most well studied to date. Yet the lncRNAs and piRNAs are certain to contribute to the setting of ischemia and neuroprotection, and there are several important future lines of investigation for all ncRNA studies. The foregoing studies highlight the difficulty of analyzing miRNA expression data, due to the diversity of ischemia and neuroprotection models, the time points examined, and the fact that since miRNAs can act in concert to exert effects on target mRNAs, focus on a single miRNA is unlikely to realistically capture endogenous responses. Thus, the first such studies must focus on ways to meaningfully translate the outcome of multiple expression profiling studies from diverse labs in order to identify global signaling pathways or targets that have been underappreciated to date. To address this issue, Xiao and colleagues sought to model complex interactions in order to identify synergistic effects within miRNA responses to brain ischemia (119). miRNA array expression data submitted to the Gene Expression Omnibus database from multiple studies in different labs were examined to identify mouse brain miRNAs differentially expressed between ischemia and control. Common predicted targets of the miRNAs were then used as a candidate subset to identify functional modules. An miRNA functional synergistic network, constructed by assembling all miRNA synergistic pairs, revealed 51 differentially expressed miRNAs identified between ischemia and control (32 upregulated and 19 downregulated). Among them, 24 miRNAs commonly regulating at least one target gene were used to construct a network. Subsequent analyses revealed 16 miRNAs forming 20 miRNA interaction pairs that participated in inflammatory responses, suggesting these 16 miRNAs as underlying targets for prevention and/or treatment of stroke (119). These analyses highlight that the power of global miRNA and ncRNA studies lies in their potential to identify proteins or pathways involved in responses to ischemia. The second such studies must address the need to identify the effects of ncRNAs acting in concert to regulate cellular phenotypes or responses to stress. To date, each family of ncRNA has largely been examined individually, due to technical barriers to combinatorial analysis of all ncRNAs. RNAseq studies can be used to identify all RNA species in a given sample set, but even this method is limited in the ability to analyze distinct sizes of RNA species. Thus, lncRNAs (>200 nt) are not yet typically sequenced together with small ncRNAs such as the miRNAs and piRNAs (~20–36 nt). A third line of exciting studies will come from the not-so-recent finding that extracellular RNAs exist in biofluids, mostly contained within EVs such as microvesicles, and can serve as biomarkers of neurological disorders (120). EVs and their molecular components are increasingly shown to be effectors of cell–cell communication in the brain and their uptake can alter the phenotype of a recipient cell (32). Thus, future lines of investigation should include examining the consequences of disrupted blood flow and metabolism, such as during a stroke, on communication mediated by EVs. Further, given that EVs mediate communication between distinct cell types in the brain, future therapeutic strategies for the treatment or prevention of stroke and other brain disorders might include focused delivery of molecular or pharmaceutical agents by EVs, potentially using vesicles isolated from ones own biofluids. The importance and excitement of such studies is highlighted by the recent formation of the Extracellular RNA Communication program (http://commonfund.nih.gov/Exrna/index) supported by the National Institutes of Health Common Fund that aims to discover fundamental biological principles about the mechanisms of exRNA generation, secretion, and transport; to identify and develop a catalog of exRNA in normal human body fluids; and to investigate the potential for using exRNAs as therapeutic molecules or biomarkers of disease. Thus, there are many new and exciting future opportunities for research and development in the field of ncRNAs, and in particular for their role in, contribution to, and regulation of the treatment of human diseases, including stroke.

## Conflict of Interest Statement

The author declares that the research was conducted in the absence of any commercial or financial relationships that could be construed as a potential conflict of interest.
